# IFITM3 Interacts with the HBV/HDV Receptor NTCP and Modulates Virus Entry and Infection

**DOI:** 10.3390/v14040727

**Published:** 2022-03-30

**Authors:** Massimo Palatini, Simon Franz Müller, Michael Kirstgen, Silke Leiting, Felix Lehmann, Lena Soppa, Nora Goldmann, Christin Müller, Kira Alessandra Alicia Theresa Lowjaga, Jörg Alber, Giuliano Ciarimboli, John Ziebuhr, Dieter Glebe, Joachim Geyer

**Affiliations:** 1 Institute of Pharmacology and Toxicology, Faculty of Veterinary Medicine, Justus Liebig University Giessen, 35392 Giessen, Germany; massimo.palatini@vetmed.uni-giessen.de (M.P.); simon.mueller@vetmed.uni-giessen.de (S.F.M.); michaelkirstgen@web.de (M.K.); silke.leiting@vetmed.uni-giessen.de (S.L.); kira.lowjaga@web.de (K.A.A.T.L.); joerg.alber@vetmed.uni-giessen.de (J.A.); 2 Institute of Medical Virology, National Reference Center for Hepatitis B Viruses and Hepatitis D Viruses, German Center for Infection Research (DZIF, Partner Site Giessen-Marburg-Langen), Justus Liebig University Giessen, 35392 Giessen, Germany; felix.lehmann@viro.med.uni-giessen.de (F.L.); lena.soppa@viro.med.uni-giessen.de (L.S.); nora.goldmann@viro.med.uni-giessen.de (N.G.); christin.mueller@bio.uni-giessen.de (C.M.); john.ziebuhr@viro.med.uni-giessen.de (J.Z.); dieter.glebe@viro.med.uni-giessen.de (D.G.); 3 Experimental Nephrology, Medical Clinic D, Muenster University Hospital, 48149 Muenster, Germany; gciari@uni-muenster.de

**Keywords:** HBV, HDV, infection, NTCP, IFITM3, virus entry, entry inhibitor, protein–protein interaction, bile acid transport, preS1 peptide

## Abstract

The Na^+^/taurocholate co-transporting polypeptide (NTCP, gene symbol *SLC10A1*) is both a physiological bile acid transporter and the high-affinity hepatic receptor for the hepatitis B and D viruses (HBV/HDV). Virus entry via endocytosis of the virus/NTCP complex involves co-factors, but this process is not fully understood. As part of the innate immunity, interferon-induced transmembrane proteins (IFITM) 1–3 have been characterized as virus entry-restricting factors for many viruses. The present study identified IFITM3 as a novel protein–protein interaction (PPI) partner of NTCP based on membrane yeast-two hybrid and co-immunoprecipitation experiments. Surprisingly, IFITM3 knockdown significantly reduced in vitro HBV infection rates of NTCP-expressing HuH7 cells and primary human hepatocytes (PHHs). In addition, HuH7-NTCP cells showed significantly lower HDV infection rates, whereas infection with influenza A virus was increased. HBV-derived myr-preS1 peptide binding to HuH7-NTCP cells was intact even under IFITM3 knockdown, suggesting that IFITM3-mediated HBV/HDV infection enhancement occurs in a step subsequent to the viral attachment to NTCP. In conclusion, IFITM3 was identified as a novel NTCP co-factor that significantly affects in vitro infection with HBV and HDV in NTCP-expressing hepatoma cells and PHHs. While there is clear evidence for a direct PPI between IFITM3 and NTCP, the specific mechanism by which this PPI facilitates the infection process remains to be identified in future studies.

## 1. Introduction

The Na^+^/taurocholate co-transporting polypeptide (NTCP, gene symbol *SLC10A1*) represents a hepatic bile acid carrier responsible for bile acid uptake from the portal blood after its intestinal reabsorption [1,2,3]. In addition, NTCP serves as the high-affinity receptor for the hepatitis B and D viruses (HBV/HDV) in the liver [4,5]. The DNA genome of HBV encodes three envelope proteins: small (SHBs), middle (MHBs), and large (LHBs). The 2–48 N-terminal amino acids of LHBs form the myristoylated preS1 domain (myr-preS1_2–48_ lipopeptide), which is essential for virus binding to NTCP [6,7]. As HDV is a satellite virus of HBV, it shares its envelope proteins with HBV and is thought to employ the same mechanism of entry into hepatocytes via NTCP as established for HBV. After the binding of HBV/HDV to the surface of hepatocytes by low-affinity interaction with heparan sulfate proteoglycans, high-affinity binding to NTCP is mandatory for virus entry [4,5]. However, the exact mechanism by which the virus/NTCP-receptor complex undergoes endocytosis to enter cells remains unclear. A number of host factors involved in this process have previously been identified, including the epidermal growth factor receptor (EGFR), which was shown to be an entry co-factor that triggers the internalization of HBV [8], and E-cadherin, a cell adhesion protein that was identified as an interaction partner of NTCP that modulates its localization in the plasma membrane and thus its virus receptor function [9]. Normally, enveloped viruses that enter host cells through endocytosis reach the cytoplasm through early endosomes, late endosomes, or endolysosomes [10]. Together with the plasma membrane, these compartments are the typical localization sites of the interferon-induced transmembrane protein 3 (IFITM3) [11], which was identified as a novel NTCP cofactor in the present study.

The IFITM protein family was first discovered in 1984 by a cDNA screen of IFN-stimulated neuroblastoma cells [12] and currently consists of five members in humans (IFITM1, 2, 3, 5, and 10), of which only IFITM1, 2, and 3 have been identified as potent inhibitors of virus entry [13,14]. IFITM proteins impair the entry of non-enveloped and enveloped RNA and DNA viruses, including influenza A virus (IAV), hepatitis C virus (HCV), dengue virus, vesicular stomatitis virus (VSV), human immunodeficiency virus type I (HIV), severe acute respiratory syndrome coronavirus (SARS-CoV), Ebola virus (EBOV), Marburg virus (MARV), and Zika virus (ZIKV) [15,16,17,18,19,20,21,22]. IFITM expression is regulated either by the interferon-stimulated response element (ISRE) in response to interferon types I and II or in an interferon-independent manner [23,24]. IFITM1-3 are all sorted to cellular membranes, with IFITM1 being predominantly localized to the plasma membrane and IFITM2-3 being mainly concentrated in endolysosomal membranes [14]. This is of particular relevance to the present study because early and late endosomes were previously identified as the sites for HBV entry and fusion [25].

The exact antiviral mechanism by which IFITMs reduce the viral entry of so many different viruses is not fully understood. However, several studies suggest a blockage of the formation of fusion pores following virus–endosome hemifusion [15,16]. One study has additionally shown that the expression rate of IFITMs in endosomes strongly correlates with the traffic time of IAV-containing endosomes to lysosomes for degradation. More precisely, Spence et al. (2019) showed that, in IFITM2/3-knockout cells, IAV-containing endosomes traffic more slowly to lysosomes by approximately 10 min compared to cells that express IFITM2 and 3 [26]. In 2018, a proteomics study was published presenting a list of IFN-dependent proteins that are upregulated during an HBV infection, listing IFITM3 as one of the most upregulated candidates [27]. Moreover, IFITM3 was shown to be upregulated upon the incubation of hepatocytes with the myr-preS1 lipopeptide, which mimics HBV/HDV binding to NTCP [28]. The present study identified IFITM3 as a novel protein–protein interaction partner of NTCP and analyzed the role of IFITM3 for NTCP expression and its receptor function for HBV/HDV.

## 2. Materials and Methods

### 2.1. Chemicals

All the chemicals, unless otherwise stated, were purchased from Sigma-Aldrich (St. Louis, MO, USA). Radio-labelled [^3^H]taurocholic acid ([^3^H]TC, 10 Ci/mmol) was purchased from PerkinElmer Life Sciences (Waltham, MA, USA).

### 2.2. Yeast Two-Hybrid Membrane Protein System (MYTH)

The yeast-two hybrid system is a method to identify protein interactions by fusion of C- and N-terminal ubiquitin resulting in the formation of split-ubiquitin [29]. Therefore, the proteins of interest must be fused either to the C-terminal unit (C_Ub_/bait protein) or to the N-terminal unit (N_Ub_/prey protein). Due to the membrane topology of NTCP with an intracellular C-terminus and IFITM3 with an intracellular N-terminus, NTCP was cloned into the bait vector pBT3-STE (C-terminal C_Ub_-LexA-VP16), while IFITM3 was cloned into the prey vector pPR3-N (N-terminal N_Ub_-HA) using *Sfi*I restriction sites. Vectors and the yeast strain NMY51 (MATa his3∆200 trp1-901 leu2-3112 ade2 LYS2::(lexAop)4-HIS3 ura3::(lexAop)8-lacZ ade2::(lexAop)8-ADE2 GAL4) were acquired from DUALsystems Biotech AG (Schlieren, Switzerland). The bait vectors feature a kanamycin resistance gene for the efficient selection of *E. coli* as well as a leucine synthesis gene for the selection of yeast cells. On the contrary, the prey vectors contain an ampicillin resistance gene for the selection of *E. coli* and a tryptophan synthesis gene for the selection of yeast cells. The successful transformation of yeast cells with both bait and prey constructs was determined by plating and growing yeast cells on synthetically defined (SD) agar plates lacking leucine and tryptophan (transformation control) and SD plates lacking leucine, tryptophan, adenine, and histidine (interaction control).

### 2.3. Transformation of NMY51 with Bait-NTCP

Yeast cells of the strain NMY51 were grown on yeast extract peptone adenine dextrose (YPAD) plates containing 1% yeast extract (Roth, Karlsruhe, Germany), 2% tryptone/peptone ex casein (Roth, Karlsruhe, Germany), 2% glucose monohydrate (Roth, Karlsruhe, Germany), 2% agar-agar Kobe I (Roth, Karlsruhe, Germany), and 0.004% adenine sulfate at 30 °C, after which multiple colonies were inoculated in 50 mL of YPAD medium prepared with the same ingredients as the YPAD plates without agar-agar and incubated at 30 °C with shaking for 16 h. The culture was then diluted with YPAD medium to an OD_600_ of 0.4 and incubated at 30 °C with shaking until an OD_600_ of 0.8 was reached. Then, the culture was pelletized and re-suspended with 2.5 mL water, and 100 µL were mixed with 300 µL polyethylene glycol (PEG)/lithium acetate (LiOAc), with the master mix containing 2.4 mL 50% PEG 4000 (Roth, Karlsruhe, Germany), 360 µL 1 M lithium acetate (Roth, Karlsruhe, Germany), and 250 µL single stranded carrier DNA (ssDNA) for 10 reactions. After addition of 1.5 µg bait-NTCP plasmid DNA (pDNA) the reaction was incubated at 42 °C for 45 min. The yeast cells were then pelletized, re-suspended with 100 µL of 0.9% NaCl, and plated onto an SD-L (minus leucine) agar plate. After 3 days, a few colonies were inoculated each in 10 mL SD-L medium and incubated at 30 °C with shaking for 16 h, after which 850 µL of the culture were mixed with 150 µL glycerol and stored at −80 °C. Expression verification of bait-NTCP in the yeast cells was already described by our laboratory before [30,31].

### 2.4. Yeast Two-Hybrid NTCP—pPR3-N Pilot Screen

Prior to the cDNA library screen, a pilot screen for determining the best concentration of 3-aminotriazole (3-AT) to keep unspecific interactions of bait-NTCP to a minimum on the selective plates was performed. Bait-NTCP transformed yeasts were plated on an SD-L agar plate, and fresh colonies were then inoculated in 10 mL SD-L medium for overnight growth at 30 °C with shaking. Then, the entire culture was transferred to 100 mL of SD-L medium and again grown overnight under the same conditions. The yeast cells were pelletized and inoculated in 150 mL of 2xYPAD medium to an OD_600_ of 0.15. Afterwards, the culture was incubated at 30 °C with shaking until an OD_600_ of 0.6 was reached (two cell divisions). The culture was then split up in 3 parts; each was pelletized and re-suspended in 1 mL LiOAc/Tris-HCl-EDTA (TE) master mix (Sigma-Aldrich, St. Louis, MO, USA) containing 0.88 mL 1 M LiOAc, 0.88 mL 10xTE (pH 7.5), and 6.24 mL double distilled water (ddH_2_O) for a total of 8 mL prior to be pelletized again and re-suspended in 600 µL LiOAc/TE master mix. The yeast cells were then added to 100 µL ssDNA, 7 µg pPR3-N pDNA (empty prey vector), and 2.5 mL PEG/LiOAc master mix (Sigma-Aldrich, St. Louis, MO, USA) containing 1.2 mL 1 M LiOAc, 1.2 mL 10xTE (pH 7.5), and 9.6 mL 50% PEG for a total of 12 mL. The reactions were incubated for 45 min at 30 °C prior to adding 160 µL dimethyl sulfoxide (DMSO) to each reaction followed by another incubation step for 20 min at 42 °C. Then, each yeast pellet was re-suspended with 3 mL of 2xYPAD, pooled, and incubated for 90 min at 30 °C with shaking. Finally, the pooled yeast pellet was re-suspended with 3.6 mL of 0.9% NaCl, and 300 µL was plated on 150 mm diameter SD-AHLW plates containing 0–30 mM 3-AT, while 100 µL of the dilutions 1:100, 1:1000, and 1:10,000 were plated on 100 mm diameter SD-LW plates for calculation of the transformation efficiency. All plates were incubated for 3 days at 30 °C.

### 2.5. Yeast Two-Hybrid NTCP—N_Ub_G-X Human Kidney cDNA Library Screen

Bait-NTCP transformed yeast cells were plated and grown in SD-L medium as described in the pilot screen. Then, the culture was transferred to 200 mL of 2xYPAD to an OD_600_ of 0.15 and all further steps were performed as described in the pilot screen, except for the split into 4 equal cultures and the use of 7 µg human kidney cDNA library pDNA (DUALsystems Biotech AG, Schlieren, Switzerland) instead of the empty prey vector pPR3-N for the yeast transformation. Finally, the transformed yeast pellet was re-suspended in 4.8 mL 0.9% NaCl and plated on 150 mm diameter SD-AHLW plates containing 25 mM 3-AT, while 100 µL of the dilutions 1:100, 1:1000, and 1:10,000 were plated on 100 mm diameter SD-LW plates.

### 2.6. Isolation and Characterization of the Interacting Prey Constructs

For the isolation of the pDNA in yeast, 200 transformed yeast colonies were inoculated in 4 mL SD-LW medium each and grown overnight at 30 °C with shaking. The plasmid was then isolated using the QIAGEN plasmid miniprep kit (QIAGEN, Hilden, Germany). For the lysis of the yeast cells, an additional step was introduced, by which glass beads were used to break the yeast cell wall. The isolated yeast pDNA was then transformed into TOP 10 chemically competent *E. coli*, which were plated on LB agar plates containing 100 µg/mL ampicillin for the selection of the prey plasmid. After the isolation of the bacterial pDNA, it was sent for sequencing of the gene of interest. The sequences were analyzed using BLAST (blast.ncbi.nlm.nih.gov, accessed on 18 December 2018). Out of 200 isolated DNA sequences, all genes with a frequency of at least 1% were visualized as an interaction map on STRING [32] and exported to the program Cytoscape (Institute for Systems Biology, Seattle, WA, USA) [33].

### 2.7. Yeast Two-Hybrid 1:1 Interaction

As described above, for the validation of the interaction between NTCP and IFITM3 in yeast, the open reading frame (ORF) of IFITM3 was cloned into the pPR3-N vector. Fresh yeast colonies were inoculated in 50 mL of YPAD medium and incubated at 30 °C overnight with shaking at 180 rpm. The culture was diluted to an OD_600_ of 0.4 and incubated at 30 °C with shaking until an OD_600_ of 0.8 was reached. The culture was pelletized and re-suspended with 2.5 mL ddH_2_O. Co-transformations were performed by using 1.5 µg of each plasmid, 300 µL PEG/LiOAc master mix (consisting of 2.4 mL 50% PEG 4000, 360 μL 1 M LiOAc, and 250 μL ssDNA for 10 reactions), and 100 µL of the yeast culture. All reactions were incubated at 42 °C for 45 min prior to pelleting and resuspension with 100 µL of 0.9% NaCl each. Then, 4 µL of the re-suspended yeast cells were dripped onto SD-LW plates for transformation control and another 4 µL on SD-AHLW plates for interaction control. All plates were incubated at 30 °C for 5 days.

### 2.8. Cell Lines

Human hepatoma HepG2 Tet-On cells (BD Clontech, Heidelberg, Germany), further referred to as HepG2 cells, were cultured in DMEM (Gibco, Carlsbad, CA, USA) and supplemented with 10% fetal calf serum (FCS, Pan-Biotech, Aidenbach, Germany), L-glutamine (4 mM, anprotec, Bruckburg, Germany), penicillin (100 U/mL, anprotec, Bruckburg, Germany), and streptomycin (100 μg/mL, anprotec, Bruckburg, Germany) in a 5% CO_2_ atmosphere at 37 °C. HepG2 cells stably transfected with tetracycline-inducible NTCP-FLAG, further referred to as HepG2-NTCP, were cultured under the same conditions. For the induction of NTCP-FLAG expression, cells were incubated with 5 µg/mL doxycycline. Human hepatoma HuH7-FlpIn cells were created by the stable transfection of HuH7 cells with the pFRT/lacZeo vector using FuGENE 6 (Roche, Basel, Switzerland) according to the manufacturer’s protocol. This vector contains the Flp Recombination Target (FRT) site and a zeocin resistance gene, which was used for the selection of successfully transfected cell clones. In a next step, these HuH7-FlpIn cells were stably transfected with the pcDNA5/FRT/TO vector (Thermo Fisher Scientific, Waltham, MA, USA) containing the ORF of human NTCP with an N-terminal HA-tag and a C-terminal FLAG-tag, as described before [34]. After transfection, cells were maintained for several weeks under a selection medium containing hygromycin (150 µg/) mL until single colonies were isolated, cultured, and tested for NTCP expression. These HuH7 cells stably transfected with HA-NTCP-FLAG are further referred to as HuH7-NTCP.

### 2.9. SiRNA Transfection

All experiments, including the knockdown of IFITM3, were performed by transfecting cells with either negative control siRNA (Thermo Fisher Scientific, Waltham, MA, USA, Cat#AM4635) or IFITM3 siRNA (Thermo Fisher Scientific, Waltham, MA, USA, Cat#AM16706). Cells were seeded on 24-well plates, except for the surface biotinylation assay, which was performed by transfecting cells seeded on 6-well plates. For the assays on 24-well plates, cells were first seeded on collagen-coated plates. For each well, a dilution of 100 pmol siRNA in 50 µL optiMEM (Gibco, Carlsbad, CA, USA) and a dilution of 2 µL Lipofectamine2000 (Thermo Fisher Scientific, Waltham, MA, USA) in 50 µL optiMEM was prepared, mixed, and then incubated at room temperature for 20 min. After the incubation time, 100 µL of the mixture was dripped on each respective well. For the transfection of cells on 6-well plates, a mixture containing a dilution of 500 pmol siRNA in 100 µL optiMEM and 10 µL Lipofectamine2000 in 100 µL optiMEM was used, out of which 200 µL were dripped on the respective wells after an incubation time of 20 min.

### 2.10. Co-Immunoprecipitation (co-IP)

The following steps were all performed on ice, unless stated otherwise. HepG2 or HuH7 cells growing on 6-well plates were washed with ice-cold phosphate-buffered saline (PBS, containing 137 mM NaCl, 2.7 mM KCl (Roth, Karlsruhe, Germany), 1.5 mM KH_2_PO_4_ (Roth, Karlsruhe, Germany), 7.3 mM Na_2_HPO_4_ (Roth, Karlsruhe, Germany), pH 7.4) and then harvested using 100 µL co-immunoprecipitation (co-IP) lysis buffer containing 20 mM Tris-HCl (Roth, Karlsruhe, Germany), 135 mM NaCl (Roth, Karlsruhe, Germany), 10% glycerol (Roth, Karlsruhe, Germany), and 1% Nonidet P40 (Fluka, Buchs, Switzerland). Cells were incubated for 60 min before centrifugation at 10,000× *g* for 10 min at 4 °C. The supernatants were transferred to new tubes, and the protein levels were measured via BCA Protein Assay Kit (Novagen, St. Louis, MO, USA). For the IP, 30 µL of Pierce Anti-Flag Magnetic Agarose (Invitrogen, Carlsbad, CA, USA) was added to 300 µg of protein lysate and incubated for 3 h at 4 °C on a rotation stand. After incubation, the agarose was washed 3 times using co-IP lysis buffer and afterwards re-suspended within 50 µL of Laemmli sample buffer containing 2% SDS (Roth, Karlsruhe, Germany), 10% glycerol (Roth, Karlsruhe, Germany), 0.002% bromophenol blue (Merck, Darmstadt, Germany), 62.5 mM Tris-HCl (Roth, Karlsruhe, Germany), and 5% 2-mercaptoethanol (Roth, Karlsruhe, Germany). Samples were then heated at 95 °C for 10 min, and the supernatants were transferred to new tubes for Western blotting.

### 2.11. Surface Biotinylation

The following steps were all performed on ice, unless stated otherwise. HepG2 or HuH7 cells were plated on collagen-coated 6-well plates and transiently transfected with siRNA as described above. Then, 72 h after transfection, cells were incubated for 15 min, and the following solutions were pre-chilled. After a washing step with PBS, cells were incubated with 1 mg/mL NHS-SS-Biotin (Thermo Fisher Scientific, Waltham, MA, USA, Cat#PG82077) in PBS with shaking. Cells were washed again with PBS and incubated with 100 mM glycine in PBS for 20 min under shaking. After another washing with PBS, cells were lysed, and the protein samples were prepared as described above, with the exception that for this assay streptavidin beads (Thermo Fisher Scientific, Waltham, MA, USA, Cat#88817) were used.

### 2.12. Western Blotting

The protein samples were loaded on 12% SDS polyacrylamide gels and transferred to nitrocellulose membranes (GE Healthcare, Buckinghamshire, UK). After blocking with 5% low-fat powdered milk (Roth, Karlsruhe, Germany) in TBS-T (consisting of 137 mM NaCl, 10 mM Tris (Roth, Karlsruhe, Germany) pH 8.0, and 0.05% Tween-20 (Roth, Karlsruhe, Germany)) for 1 h, the membranes were incubated with the primary antibody in blocking solution at 4 °C overnight. After three washing steps in TBS-T, the membranes were incubated with the secondary antibody in blocking solution at room temperature for 1 h. After three washing steps, target proteins were visualized by chemiluminescence imaging (ChemiDoc, BioRad, Hercules, CA, USA). Band quantification was performed with the Image Lab software (BioRad, Hercules, CA, USA) on the original images. For this, the respective FLAG/NTCP or IFITM3 bands were quantified and set in relation to glyceraldehyde-3-phosphate dehydrogenase (GAPDH). The following primary antibodies were used: anti-FLAG (rabbit, 1:2000; Sigma-Aldrich, St. Louis, MO, USA, Cat#F7425), anti-IFITM3 (rabbit, 1:5000; Proteintech, Rosemont, IL, USA, Cat#11714), anti-SLC10A1 (rabbit, 1:1000; Sigma-Aldrich, St. Louis, MO, USA, Cat#HPA042727), and anti-GAPDH (goat, 1:1000; Sigma-Aldrich, St. Louis, MO, USA, Cat#SAB2500450). The following secondary antibodies were used: anti-rabbit-HRP (goat, 1:4000; Thermo Fisher Scientific, Waltham, MA, USA, Cat#31460) and anti-goat-HRP (rabbit, 1:5000; Thermo Fisher Scientific, Waltham, MA, USA, Cat#81-1620).

### 2.13. HBV Infection of HepG2-NTCP and HuH7-NTCP Cells

Cells were infected with cell-culture-produced HBV (subgenotype D3) derived from a stably transfected inducible HepG2 cell line. The viral load was determined via qPCR as described [35]. Infection was carried out with 5 × 10^9^ HBV genomes/well of a 24-well plate. HepG2-NTCP and HuH7-NTCP cells were infected and maintained post-infection in hepatocyte growth medium (HGM) containing either 1% (HuH7-NTCP) or 2% DMSO (HepG2-NTCP) and caspofungin (5 µg/mL final concentration (f.c.))/fungizone (1 µg/mL f.c.) [36]. For infection, HGM was additionally supplemented with 4% PEG, and cells were incubated with inoculum overnight, three days after siRNA transfection. After infection, media were renewed every second day until supernatants were collected on day 10 post-infection. HBeAg secreted by infected cells was determined using the qualitative HBeAg Architect assay (Abbott, Wiesbaden, Germany).

### 2.14. Production of Pseudotyped HDV Particles

HuH7 cells were transfected with equimolar amounts of the pcDNA3.1(+) expression plasmid encoding a dimer of the respective HDV genome CHD_WHO (genotype 1; KY495779.1 [37]) or pHDV-1T (genotype 1; M21012.1 [38]) and the expression plasmid encoding the hepadnaviral L-ORF (subgenotype D3, see above) using FuGENE HD Transfection Reagent (Promega, Madison, WI, USA) according to the manufacturer’s protocol. After transfection, cells were cultivated in William’s E Medium (Thermo Fisher Scientific, Waltham, MA, USA) supplemented with 10 mM HEPES, 1× GlutaMAX Supplement (Thermo Fisher Scientific, Waltham, MA, USA), 100 units/mL of penicillin, 100 µg/mL of streptomycin, and 2% FCS. The medium was changed every 2–3 days. Supernatants from day 3 to day 13 were collected and concentrated by membrane ultrafiltration (VIVASPIN 20 PES, 300,000 MWCO; Sartorius Stedim Lab Ltd., Stonehouse, UK) according to the manufacturer’s instructions and subsequently used for infection assays. HDV viral load within the concentrates was quantified by determining the number of international units by a specific in-house RT-qPCR.

### 2.15. HDV Infection of HepG2-NTCP and HuH7-NTCP Cells

Infection of HepG2-NTCP and HuH7-NTCP with cell-culture-produced HDV bearing an HBV viral envelope was performed three days after siRNA transfection using 2–4 × 10^5^ HDV IU/well of a 24-well plate. Post-infection, cells were maintained in HGM containing either 1% (HuH7-NTCP) or 2% DMSO (HepG2-NTCP) and caspofungin (5 µg/mL f.c.)/fungizone (1 µg/mL f.c.). For infection, HGM was additionally supplemented with 4% PEG, and cells were incubated with inoculum overnight. After infection, media were renewed every 2–3 days until cells were fixed, permeabilized using 4% paraformaldehyde/0.2% Triton-X-100 in PBS for 1 h, and blocked by incubation with 10% FCS in PBS for 30 min on day 8 post-infection. Cells were immunostained with human anti-HDV-positive serum and anti-human-IgG secondary antibody coupled to Alexa Fluor 568, as previously described [39]. To determine the total cell count, nuclei were stained with DAPI. Subsequently, the percentage of HDV-infected cells was analyzed using the ImageXpress Pico automated cell imaging system (Molecular Devices LLC, San Jose, CA, USA).

### 2.16. HBV and HDV Infection of Primary Human Hepatocytes

Primary human hepatocytes (PHH, Primacyt, Schwerin, Germany) were maintained in human hepatocyte maintenance medium (HHMM; Primacyt, Schwerin, Germany) supplemented with 1.5% DMSO and caspofungin (5 µg/mL f.c.)/fungizone (1 µg/mL f.c.). Infection was performed with 5 × 10^9^ HBV genomes/well or, accordingly, 4 × 10^5^ HDV IU/well of a 24-well plate. For infection, HHMM was additionally supplemented with 4% PEG, and infection was carried out overnight, two days after siRNA transfection. The mye-preS1_2–48_ peptide, corresponding to HBV genotype D (Bio-Synthesis, Lewisville, TX, USA), was added to the medium starting half an hour before infection and until 3 days after infection. Cell culture media were exchanged every day and collected every second day. HBV and HDV infected cells were measured as described above. 

### 2.17. Influenza A Virus Infection of HuH7-NTCP Cells

HuH7-NTCP cells were transfected with siRNA as described above. Then, 72 h after transfection, cells were washed with PBS and infected with IAV (A/Thailand/1 (KAN-1)/2004) with an MOI of 0.5. After 1 h of inoculation, the medium was exchanged with DMEM containing 0.2% bovine serum albumin (BSA), 100 U penicillin, 0.1 mg/mL streptomycin, and TPCK-treated trypsin and further incubated at 37 °C for 24 h. Afterwards, cells were washed twice and fixed with freshly prepared 3.4% formaldehyde + 0.1% Triton X-100. Immunostaining was performed using mouse anti-IAV-nucleoprotein (NP) mAb (1:100, kindly provided by S. Ludwig, Münster, Germany) and goat anti-mouse IgG (H+L) secondary antibody conjugated with Alexa Fluor 594 (1:500; Thermo Fisher Scientific, Waltham, MA, USA, Cat# A-11032). Analysis of the relative number of virus-positive cells, judged by NP-signal, was performed using the ImageXpress Pico (Molecular Devices, San Jose, CA, USA) automated cell imaging system.

### 2.18. Transport Experiments

Cells were seeded on 24-well plates, transfected with siRNA as described above, and grown to confluence over 72 h at 37 °C. Afterwards, cells were washed three times with PBS prior to pre-incubation with transport buffer (consisting of 142.9 mM NaCl, 4.7 mM KCl, 1.2 mM MgSO_4_ (Roth, Karlsruhe, Germany), 1.2 mM KH_2_PO_4_, 1.8 mM CaCl_2_ (Roth, Karlsruhe, Germany), and 20 mM HEPES (Roth, Karlsruhe, Germany), pH 7.4, 37 °C) for 5 min. For uptake experiments with HepG2-NTCP and HuH7-NTCP cells, cells were incubated with 300 µL transport buffer containing 10 µM taurocholic acid (TC) spiked with [^3^H]TC for 10 min at 37 °C. For uptake experiments with PHHs, cells were incubated with 300 µL transport buffer containing 20 µM TC spiked with [^3^H]TC for 15 min at 37 °C. Serving as controls for specific NTCP-dependent TC uptake, some wells with PHHs were preincubated with 500 nM preS1-peptide (as NTCP specific inhibitor) prior to [^3^H]TC uptake or incubated in sodium-free transport buffer during [^3^H]TC uptake (to calculate NTCP dependent net uptake). Uptake reactions for all cell types were terminated by removing the transport buffer followed by five washing steps with ice-cold PBS. Then, cells were lysed in 1 N NaOH containing 0.1% SDS, and cell-associated radioactivity was measured by liquid scintillation counting in a Tri-Carb 2910 TR (PerkinElmer Life Sciences, Waltham, MA, USA). The protein content of each well was determined by Lowry assay, as reported before [34].

### 2.19. RNA Isolation and Quantitative Real-Time PCR (qRT-PCR)

Cells were lysed in RNA Lysis Buffer, and total RNA was extracted by a Quick-RNA Miniprep Kit (Zymo Research, Irvine, CA, USA). Then, 1 µg of the total RNA was reverse transcribed using SuperScript III Reverse Transcriptase (Invitrogen, Carlsbad, CA, USA). The quantitative gene expression of IFITM3 and NTCP was determined by an Applied Biosystems 7300 Real-Time PCR System using the TaqMan Gene Expression Assays Hs03057129_s1 and Hs00161820_m1 (Thermo Fisher Scientific, Waltham, MA, USA), respectively. As an endogenous control, GAPDH (Hs99999905_m1) was used. General PCR conditions were initial denaturation at 95 °C for 10 min and 40 cycles of 95 °C for 15 s and 60 °C for 60 s. Samples were run in duplicates, and relative expression was calculated as fold change using the 2^−ΔΔCt^ method.

### 2.20. PreS1 Peptide Binding Experiments

Peptide-binding experiments were performed with a tritium-labelled myr-preS1_2–48_ lipopeptide -HBV subgenotype D3- (further referred to as [^3^H]preS1) that was purchased from Pharmaron (120 Ci/mmol, 1 mCi/mL, Cardiff, UK), as reported [36]. Briefly, cells were seeded on 24-well plates, transfected with siRNA as described above, and incubated over 72 h at 37 °C. Then, cells were washed once with tempered PBS at 37 °C and preincubated with 200 µL DMEM for 5 min at 37 °C. The medium was replaced by 200 µL DMEM containing 5 nM [^3^H]preS1, and cells were further incubated for 10 min at 37 °C. Experiments were stopped by removing the medium and washing five times with ice-cold PBS. Cells were lysed in 1 N NaOH with 0.1% SDS, and cell-associated radioactivity was measured by liquid scintillation counting. The protein content of each well was determined by Lowry assay as described above.

## 3. Results

### 3.1. Protein–Protein Interaction between NTCP and IFITM3

**Figure 1:**
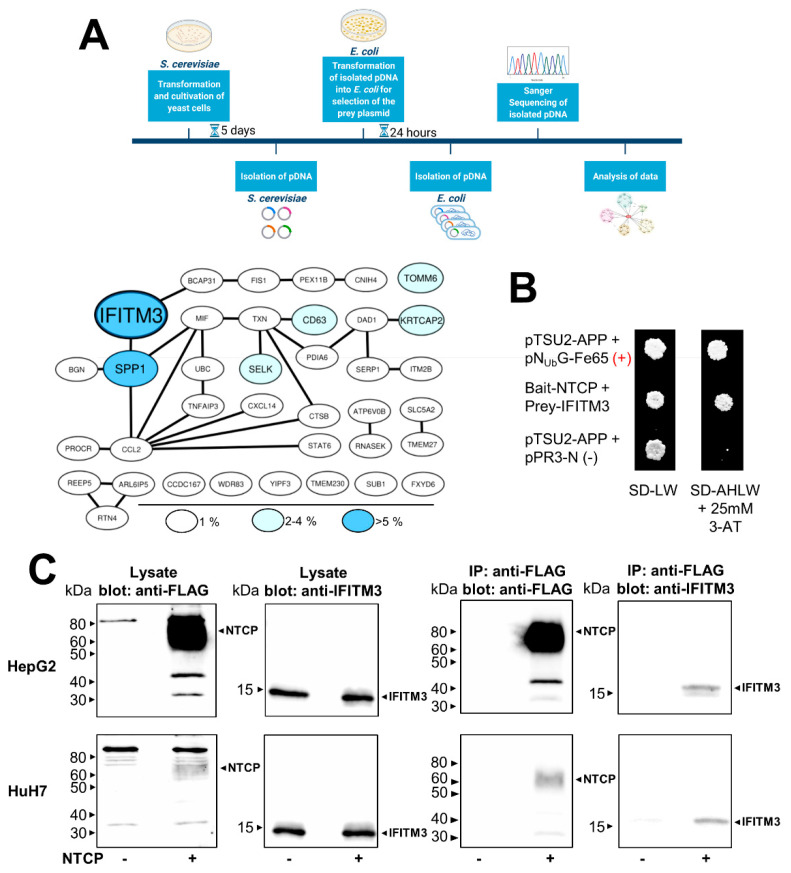
Protein–protein interaction between NTCP and IFITM3. (**A**) Yeast cells were co-transformed with the NTCP-C_Ub_-LexA-VP16 bait construct and the N_Ub_-human kidney cDNA prey library. The pDNA of 200 yeast cells grown on SD-AHLW plates was selected for the interacting prey construct by transformation of the pDNA in *E. coli* and incubation on plates containing ampicillin. The isolated prey pDNAs were sequenced, and the corresponding proteins were analyzed and illustrated in an interaction map. Color and size of the protein labels indicate the frequency among the 200 hits analyzed. IFITM3 was the most common hit with an 11/200 hit rate. (**A**) was created with BioRender (biorender.com). (**B**) The ORF of IFITM3 was sub-cloned into the pPR3-N prey vector and co-transformed with the NTCP-C_Ub_-LexA-VP16 bait construct into yeast cells. In addition, the control construct pTSU2-APP was co-transformed with either the pN_Ub_G-Fe65 prey construct (positive control) or the pPR3-N empty vector (negative control). NTCP-C_Ub_-LexA-VP16 expression from the bait construct was previously described [31]. Colony growth was analyzed on SD-LW plates (lacking leucine and tryptophan) and on SD-AHLW plates (additionally lacking adenine and histidine). (**C**) HepG2-NTCP and HuH7-NTCP cells were seeded on 6-well plates; non-NTCP-expressing HepG2-TetOn and HuH7-FlpIn cells served as negative controls. Cells were lysed after 48 h, and the lysates were used for IP with anti-FLAG agarose. The lysate and IP samples were processed for Western blotting with an anti-FLAG antibody or an anti-IFITM3 antibody.

To identify potential protein–protein interaction (PPI) partners of NTCP that might be involved in the HBV/HDV entry and infection process, a cDNA library was screened for potential NTCP interaction partners using the yeast two-hybrid membrane protein system. The screening was performed by the co-transformation of bait-NTCP with an N-terminal HA-N_Ub_G-tagged human kidney cDNA library in yeasts. The screening revealed the growth of several hundreds of colonies on the SD-AHLW plates, which only allow the growth of the yeasts when the bait-NTCP interacts with one of the proteins from the prey cDNA library. Among 200 isolated and sequenced clones, interferon-induced transmembrane protein 3 (IFITM3) was the dominant hit, with a number of 11 colonies out of 200, and the second-most-frequent hit was secreted phosphoprotein 1 (SPP1) (Figure 1A). The present study focused on the characterization of a potential PPI between NTCP and IFITM3. To verify this screening hit, a full-length human IFITM3 prey construct was de novo generated and used for the direct (1:1) interaction between bait-NTCP and prey-IFITM3 in the yeast cells. As depicted in Figure 1B, the co-transformation of bait-NTCP and prey-IFITM3 enabled colony growth on the selective SD-AHLW plates, clearly indicating the PPI of NTCP and IFITM3 in the yeasts. As additional controls, pTSU-APP was co-transformed with pN_Ub_G-Fe65 (positive control) or with pPR3-N (negative control), and unspecific interactions of prey-IFITM3 were excluded by co-transformation with the control construct pTSU2-APP, which did not show any growth of the yeasts (data not shown).

Next, the PPI between NTCP and IFITM3 was analyzed in two human hepatoma cell lines, namely HepG2 and HuH7, which were stably transfected with an NTCP-FLAG construct as reported before [4]. In these experiments, HepG2-NTCP and HuH7-NTCP cells as well as non-transfected control cells were used for co-IP experiments with anti-FLAG agarose to precipitate the NTCP-FLAG proteins. As IFITM3 is endogenously expressed in both cell lines, it was not additionally transfected for overexpression. The Western blot analysis confirmed a successful IP of NTCP-FLAG from both cell lines using anti-FLAG magnetic agarose beads. Of note, NTCP protein expression was much higher in the HepG2-NTCP cells compared to the HuH7-NTCP cells. After the immunoprecipitation of NTCP, Western blot analysis with the IFITM3 antibody revealed co-precipitation of IFITM3, clearly confirming the PPI of NTCP and IFITM3 also in hepatoma cells. NTCP expression was not detected in the non-transfected HepG2-TetOn and HuH7-FlpIn cells (NTCP “-“), and in these cells, also, no precipitation of IFITM3 was detected (Figure 1C).

### 3.2. Knockdown of IFITM3 and NTCP Expression

**Figure 2:**
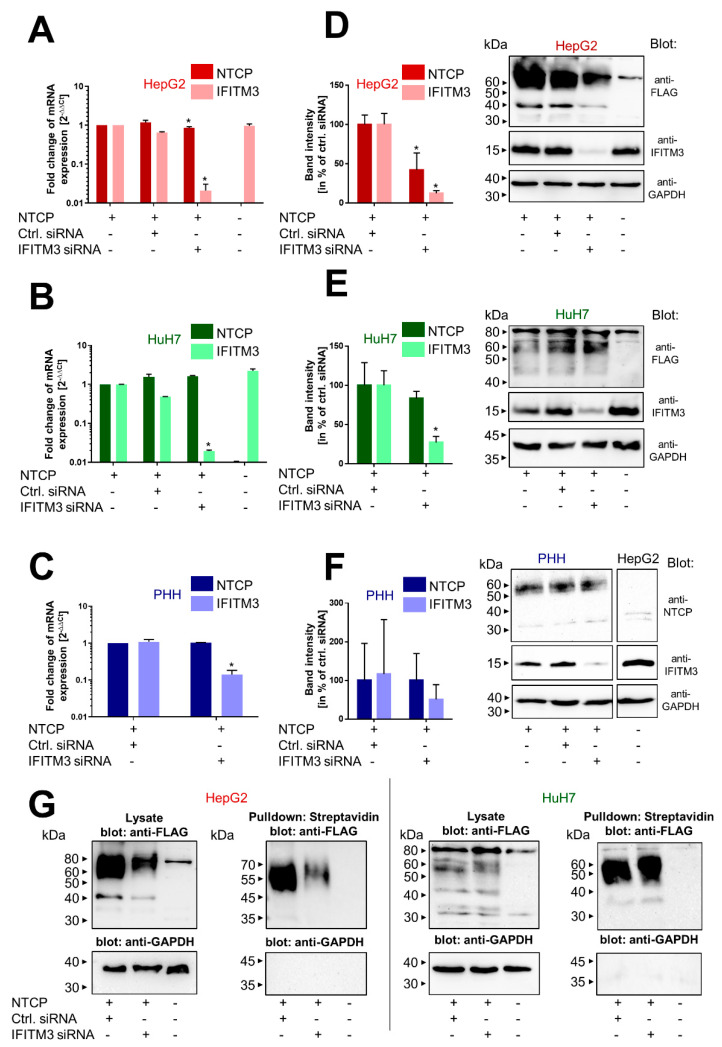
Knockdown of IFITM3 and NTCP expression. PHHs as well as NTCP-expressing and non-NTCP-expressing HepG2 and HuH7 cells were transiently transfected with either control or IFITM3 siRNA and incubated for 72 h. (**A**–**C**) Cells were lysed using RNA lysis buffer and processed for determination of the gene expression of NTCP and IFITM3 via qRT-PCR. Expression of GAPDH served as an endogenous control, and non-siRNA-transfected cells served as calibrator (set to a value of 1). Data represents means ± SD of two (HepG2 and HuH7) or three (PHH) independent experiments each with duplicate determinations (*n* = 4 and *n* = 6, respectively). * Significantly lower mRNA expression compared to control siRNA transfected cells with *p* < 0.05 according to two-way ANOVA with Sidak multiple comparisons test. (**D**–**F**) Cells were lysed using protein lysis buffer and processed for Western blotting with antibodies against FLAG, NTCP, IFITM3, or GAPDH, respectively. Band intensities were quantified by using the Image Lab software and expression of FLAG/NTCP was normalized to GAPDH, respectively. (**G**) Cells were incubated with NHS-SS-biotin prior to lysis, and pulldown with streptavidin-coupled beads was performed to separate surface proteins. All samples were processed for Western blotting using an anti-FLAG antibody and an anti-GAPDH antibody.

To analyze the importance of IFITM3 and its interaction with NTCP in the context of HBV/HDV entry and infection, a knockdown of IFITM3 was performed in the HepG2 and HuH7 hepatoma cells as well as in PHHs using an siRNA approach. The successful and significant knockdown in all cell lines was verified on the mRNA level by qRT-PCR (Figure 2A–C) and on the protein level by Western blotting (Figure 2D–G). As the only exception, IFITM3 protein expression was lower in the PHH after IFITM3 siRNA transfection, but this difference did not reach the level of significance after densitometry quantification of the Western blot signals when all four independent experiments were combined (Figure 2F). This is most likely due to a higher variability in transfection rates of primary cells compared to permanent cell lines when conventional transfection methods are used. In all knockdown experiments, cells transfected with negative control siRNA served as controls, and the control siRNA did not alter the expression of either NTCP or IFITM3. Of note, transfection of the IFITM3 siRNA induced apart from IFITM3 downregulation also significantly lower levels for NTCP mRNA and protein expression in the HepG2 cells (Figure 2A,D). This effect was not observed in the HuH7-NTCP cells and the PHHs. As Western blot analysis with the anti-FLAG antibody only allowed a conclusion about the total amount of NTCP expression in the cells irrespective of its localization within the cell, the plasma membrane expression of NTCP was additionally analyzed by surface biotinylation experiments (Figure 2G). Cells were incubated with the non-membrane permeable reagent NHS-SS-biotin so that after streptavidin pulldown, only surface proteins were separated. As expected, NTCP was detected via anti-FLAG Western blot after surface biotinylation in both hepatoma cell lines, HepG2-NTCP and HuH7-NTCP. Yet, again, lower NTCP levels were detected in the HepG2-NTCP cells after IFITM3 knockdown. In contrast, no difference in the surface biotinylation of NTCP was observed in the HuH7-NTCP cells after IFITM3-siRNA transfection.

### 3.3. Transporter and Receptor Function of NTCP under IFITM3 Knockdown

**Figure 3:**
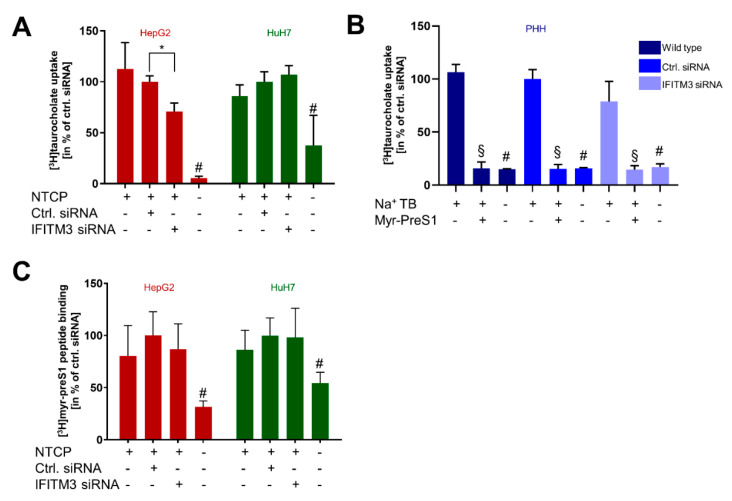
Transporter and receptor function of NTCP under IFITM3 knockdown. PHH, HepG2, and HuH7 cells were seeded on 24-well plates, transfected with either control siRNA or IFITM3 siRNA, and incubated for 72 h (**A**,**C**) or 48 h (**B**). (**A**) Cells were incubated for 10 min with 10 µM [^3^H]TC, and transport rates were determined. [^3^H]TC transport rates in the HepG2-NTCP and HuH7-NTCP cells after control siRNA transfections were set to 100%. Data represent means ± SD of combined data of three independent experiments each with quadruplicate determinations (*n* = 12). * Significantly lower compared to control siRNA-transfected cells and # significantly lower compared to all other conditions with *p* < 0.05 and according to two-way ANOVA with Sidak multiple comparisons test. (**B**) Cells were incubated for 15 min with 20 µM [^3^H]TC, and transport rates were determined. [^3^H]TC transport rates after control siRNA transfections were set to 100%. Data represent means ± SD of combined data of two independent experiments each with duplicate determinations (*n* = 4). # Significantly lower in sodium-free transport buffer and § significantly lower in the presence of myr-preS1 as NTCP inhibitor with *p* < 0.05 according to two-way ANOVA with Sidak multiple comparisons test. (**C**) Cells were incubated for 10 min with 5 nM [^3^H]preS1 and binding rates were determined after IFITM3 siRNA transfection. [^3^H]preS1 binding after control siRNA transfection was set to 100%. Data represent means ± SD of combined data of three independent experiments each with quadruplicate determinations (*n* = 12). # Significantly lower compared to all other conditions with *p* < 0.05 according to two-way ANOVA with Sidak multiple comparisons test.

In the next step, we analyzed the physiological bile acid transport function of NTCP under IFITM3 knockdown conditions. In detail, the TC transport was analyzed in HepG2-NTCP, HuH7-NTCP, and PHH cells after transfection with the control and IFITM3 siRNAs. As shown in Figure 3A, IFITM3 knockdown did not alter the TC transport rate in the HuH7-NTCP cells but significantly reduced NTCP’s transport function in the HepG2-NTCP cells. This data goes in line with the lower NTCP protein expression in the HepG2-NTCP cells after IFITM3 siRNA transfection (Figure 2D). In contrast, in PHH, IFITM3 siRNA transfection did not alter the TC transport rate (Figure 3B). In these experiments, TC transport rates were additionally analyzed under sodium-free conditions and in the presence of the HBV-derived viral myr-preS1 peptide as the NTCP inhibitor. As expected, under both conditions, the TC transport rate significantly dropped to background levels. Moreover, binding of the myr-preS1 peptide to NTCP was analyzed, as a well-established surrogate parameter for HBV/HDV virus binding to the receptor NTCP. For this purpose, cells were incubated with radio-labelled [^3^H]myr-preS1 for 10 min, and the amount of radioactivity after excessive washing and cell lysis was measured. No significant differences in the binding of myr-preS1 depending on IFITM3 knockdown were detected (Figure 3C).

### 3.4. Effect of IFITM3 Knockdown on the HBV/HDV Receptor Function of NTCP

**Figure 4:**
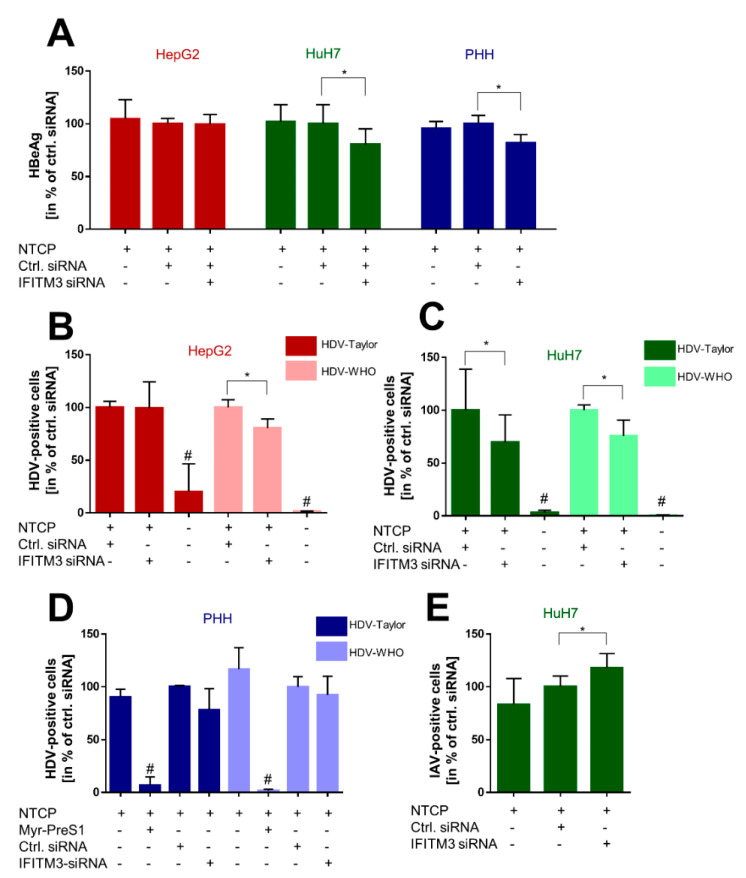
Effect of IFITM3 knockdown on the HBV/HDV receptor function of NTCP. PHH, HepG2, and HuH7 cells were seeded on 24-well plates, transfected with either control or IFITM3-siRNA, and incubated for 72 h (HepG2/HuH7) or 48 h (PHH). (**A**) Cells were inoculated with 5 × 10^9^ HBV genomes/well overnight prior to medium changes every second day until collection of supernatants on day 10 post-infection and quantification of secreted HBeAg using the HBeAg Architect assay. Data represent means ± SD of combined data from three independent experiments each with triplicate or quadruplicate determinations. * Significantly lower after IFITM3 siRNA transfection compared to control siRNA with *p* < 0.05 according to two-way ANOVA with Sidak multiple comparisons test. (**B**–**D**) Cells were inoculated with 2–4 × 10^5^ HDV IU/well overnight prior to medium changes every 2–3 days until fixation and immunostaining with human anti-HDV-positive serum on day 8 post-infection. Quantification of infected cells was performed using an automated imaging. Data represent means ± SD of combined data from three independent experiments each with triplicate determinations (**B**,**C**) or from two independent experiments with duplicate determinations (**D**). * Significantly lower after IFITM3 siRNA transfection compared to control siRNA with *p* < 0.05 according to two-way ANOVA with Sidak multiple comparisons test. # Significantly lower compared to all other conditions with *p* < 0.05 according to two-way ANOVA with Sidak multiple comparisons test. (**E**) Cells were inoculated with an MOI of 0.5 of IAV for 1 h prior to medium change. 24 h later, cells were fixated and immunostained against IAV-NP, and the number of virus-positive cells was quantified by an automated imaging. Data represent means ± SD of combined data of three independent experiments each with quadruplicate determinations. * Significantly higher after IFITM3 siRNA transfection compared to control siRNA with *p* < 0.05 according to one-way ANOVA with Tukey’s multiple comparisons test.

After this indirect approach via myr-preS1 peptide binding to NTCP, we aimed to directly analyze the effect of IFITM3 knockdown on the infection rates with HBV and HDV particles. For these experiments, both hepatoma cell lines HepG2-NTCP and HuH7-NTCP, as well as PHH, were transiently transfected with IFITM3 siRNA and control siRNA, and infection was performed by inoculation with 5 × 10^9^ HBV genomes/well or with 2–4 × 10^5^ HDV IU/well. Two different HDV strains were used, here described as HDV-1T and HDV-WHO, both representing genotype 1. HBV infection was quantified via the secretion of HBeAg, and HDV infection was analyzed based on the anti-HDV immunostaining and automated quantification of infected cells. Interestingly, all three cell types showed a unique reaction pattern after IFITM3 knockdown on in vitro HBV and HDV infections. HepG2-NTCP cells revealed no effect on HBV infection and showed significantly lower infection rates exclusively after inoculation with the HDV-WHO strain (Figure 4A,B). In contrast, HuH7-NTCP cells showed significantly lower infection rates in all assays with HBV and HDV after IFITM3 knockdown (Figure 4A,C). Finally, PHH revealed significantly lower HBeAg secretion after transfection of the IFITM3 siRNA. Additionally, the HDV-1T and HDV-WHO infection rates were lower for the IFITM3 siRNA transfected cells but without reaching the level of significance (Figure 4A,D).

To verify the role of IFITM3 on virus infections of the most reactive HuH7-NTCP cells, these cells were additionally infected with IAV. As it is well-established that IFITM3 restricts the infection with IAV, these infection experiments served as an additional control for efficient IFITM3 knockdown in the HuH7-NTCP cells. HuH7-NTCP cells were transfected with siRNA under the same conditions as in all other experiments. As expected, the knockdown of IFITM3 led to significantly higher IAV infection rates compared to the control siRNA transfected HuH7-NTCP cells (Figure 4E).

## 4. Discussion

In the present study, IFITM3 was identified as a novel NTCP co-factor that has a significant effect on the HBV and HDV entry and infection process in NTCP-expressing hepatoma cells as well as PHHs. Based on the known role of IFITM3 as the virus restriction factor, we aimed to clarify its role for HBV/HDV entry and infection. The PPI between NTCP and IFITM3 was first identified in yeast cells by MYTH screening and was then confirmed in HepG2-NTCP and HuH7-NTCP cells by co-IP (Figure 1). Next to IFITM3, SPP1 was the second-most-frequent hit from the MYTH screening. SPP1 encodes for the protein osteopontin that plays a physiological role in bone mineralization and is a regulator of inflammatory processes [40]. However, potential PPI between NTCP and osteopontin were not analyzed in detail in the present study. We decided to use three different cell culture models to analyze and corroborate this novel PPI between NTCP and IFITM3, taking into account the specific characteristics of these cell lines and leading us to more general conclusions. HepG2-NTCP cells have become a well-established cell culture model for in vitro HBV and HDV infection studies [4,36,41]. HepG2 cells have an epithelial-like morphology and are derived from a hepatocellular carcinoma. NTCP shows high expression rates in this cell type after stable transfection [4], which was confirmed by qRT-PCR and Western blot experiments in the present study. HuH7 also represents a cellular carcinoma cell line, which is most commonly used as a cell model for hepatoma and hepatitis C virus (HCV) research [42,43]. In our hands, HuH7-NTCP cells are also suitable for in vitro HBV and HDV infection; however, in comparison to HepG2-NTCP cells, NTCP overexpression is less effective. Consequently, strong Western blot signals were obtained for the NTCP-FLAG protein expressed in HepG2-NTCP cells, while only week signals were obtained for HuH7-NTCP cells that stably express NTCP-FLAG (Figure 1C and Figure 2G). This difference must be considered when interpreting the in vitro HBV/HDV infection experiments of the present study. Aside from NTCP expression levels, it should be noticed that HepG2 and HuH7 hepatoma cell lines differ in their transcriptomes and proteomes [44], which probably explains the somewhat different results between these two cell lines. As a third cell type, we used PHH for in vitro HBV and HDV infection. PHHs are the only cells that are thought to have the complete set of potential NTCP co-factors and virus entry factors and are thus considered the gold standard for in vitro HBV/HDV infection studies. PHH showed expression of the NTCP protein at a level comparable to that in HuH7-NTCP cells (Figure 2E,F). Interestingly, all three cell types differently responded to IFITM3 knockdown, which, in the present study, was used to characterize a potential role of IFITM3 in the expression and receptor function of NTCP. Significant IFITM3 knockdown was confirmed for all three cell types using quantitative real-time PCR and Western blotting. As the only exception, IFITM3 knockdown did not reach the level of significance in the PHH when four different experiments were combined (Figure 2F). This was most likely due to a certain variability of these primary hepatocytes compared to the stable HepG2-NTCP and HuH7-NTCP cells that all derive from the same cell clone and, therefore, showed lower variability when different independent experiments were combined (Figure 2D,E). The clearest data were obtained with the HuH7-NTCP cells. In these cells, IFITM3 knockdown was highly effective and did not affect NTCP expression (neither at the mRNA nor at the protein level) and bile acid transport rates (Figure 2B,E and Figure 3A). Additionally, the membrane expression of NTCP was unaffected, as shown by surface biotinylation and myr-preS1 binding experiments, which did not reveal differences between control siRNA and IFITM3 siRNA transfections (Figure 2G and Figure 3C). We could also confirm that IFITM3 knockdown significantly increased the infection rates of HuH7-NTCP cells for IAV (Figure 4E), confirming that our HuH7-NTCP cell clone is reactive in the manner reported in the literature [45]. Most interesting was the finding that HBV and HDV infection rates of HuH7-NTCP cells were significantly lower under IFITM3 knockdown conditions compared to control siRNA transfection. This data clearly indicates that IFITM3 is a novel relevant cofactor for cellular HBV/HDV virus entry and leads us to hypothesize that the molecular interaction of NTCP and IFITM3 is relevant for one of the entry steps of the virus/NTCP–receptor complex. However, based on the data obtained in this study, it is not possible to draw conclusions on whether this interaction is relevant for the induction of endocytosis and/or trafficking of the virus/NTCP-complex to certain intracellular compartments. This question must be addressed in subsequent studies. The relevance of IFITM3 for virus entry is supported by the data from the PHHs. Although PHHs showed significantly lower HBV infection rates under IFITM3 knockdown conditions, the reduction of the HDV infection rates did not reach the level of significance. This could be due to less-effective IFITM3 knockdown in PHHs compared the HuH7-NTCP cells (Figure 2C,F) or due to the higher variability between the experiments. Of note, PHHs were from the same donor but were cultivated independently from cryopreserved vials for each experiment, whereas all HuH7-NTCP and HepG2-NTCP cells were derived from permanent cell cultures. The situation is different with the HepG2-NTCP cells. In these cells, IFITM3 knockdown not only efficiently reduced the IFITM3 mRNA and protein expression (Figure 2A,D) but also reduced the total NTCP mRNA and protein expression for unknown reasons. Consequently, NTCP surface expression and bile acid transport rates were lower in HepG2-NTCP cells after IFITM3 knockdown. In addition, myr-preS1 peptide binding was lower after IFITM3 siRNA transfection compared to the control siRNA, however without reaching the level of significance, while in vitro HBV infection seemed to be unaffected. A possible explanation for this effect is that in vitro HBV infections are ineffective in the HepG2-NTCP cell culture model. Despite extremely high NTCP overexpression and massive overload with 5 × 10^9^ genomes/well for in vitro HBV infection, only a small fraction of ~10% of the cells are infected. Based on this, it seems that a relatively modest reduction of NTCP surface expression retained enough of the protein in the plasma membrane to sustain full susceptibility to in vitro HBV infection. Likewise, IFITM3 might only exert its function as relevant NTCP co-factor for virus entry at a moderate (physiological) expression level of NTCP, as was the case in the PHH and HuH7-NTCP cells, but not under conditions of massive NTCP overexpression, as was the case in HepG2-NTCP cells, even if NTCP (over)expression was reduced by IFITM3 knockdown. Based on this data, it is tempting to speculate that, in HepG2-NTCP cells, IFITM3 has a different effect on NTCP. In this situation, IFITM3 could be an NTCP-stabilizing factor in the plasma membrane, either by maintaining NTCP in a specific conformation favorable for membrane localization or by preventing NTCP from endocytosis in the absence of virus binding. This would at least explain why the knockdown of such a stabilizing factor would significantly reduce the protein expression and membrane localization of NTCP, as shown in Figure 2D,G. Based on all of the above, the data obtained from the HepG2-NTCP cells is difficult to interpret because NTCP is not retained in equal amounts in the plasma membrane and is expressed above the physiological level in this cell line. Nevertheless, the potential stabilizing role of IFITM3 on NTCP’s plasma membrane expression should also be further investigated. 

Members of the IFITM protein family have previously been identified as virus restriction factors for a broad spectrum of enveloped and non-enveloped RNA and DNA viruses [46]. However, many pathogenic viruses have evolved strategies to evade the host immune system and particularly the IFN-induced immune response, including HBV [47,48,49,50,51,52,53]. Nevertheless, PEGylated interferon-α (PEG-IFN-α) is effectively used for the treatment of chronic hepatitis B [54]. In this context, our experiments surprisingly showed a positive rather than negative effect of IFITM3 on in vitro HBV/HDV infection. At least in HuH7-NTCP and PHH cells, the siRNA-mediated knockdown of IFITM3 led to a significant decrease of HBV infection rates, whereas, in HuH7 cells, IAV infection was increased as expected and previously reported [45] (Figure 4A,E). A second surprising finding was that the infection rate of the HDV-1T strain was reduced only in HuH7-NTCP cells under IFITM3 knockdown conditions, but not in HepG2-NTCP cells, even if these cells showed reduced NTCP expression as discussed above. In contrast, reduced infection rates were detected for the HDV-WHO strain under IFITM3 knockdown in both cell lines, HepG2-NTCP and HuH7-NTCP. Of note, the HDV-1T strain was originally isolated from a patient and was passaged in a chimpanzee and a woodchuck before being cloned as a complementary DNA (cDNA) [38]. Therefore, it is unclear whether this construct authentically represents naturally circulating isolates [55]. Our data indicate that, at least in HepG2 cells, in vitro infection with the HDV-1T strain proceeds in an IFITM3-independent manner, whereas infection rates with the HDV-WHO strain were reduced in the HepG2-NTCP and HuH7-NTCP cells under IFITM3 knockdown. However, the mechanism by which IFITM3 exactly affects in vitro HBV/HDV infection via NTCP remains unclear. A previous study showed a direct interaction between IFITM3 and v-ATPase, which promotes the acidification of endosomes and causes a neutral pH in endosomes of murine IFITM-knockout cells in vitro [56]. Based on this, it seems reasonable to suggest that an endosomal pH change under IFITM3 knockdown might affect the release of HBV and HDV from the endosome compartment and thereby also the infection rates. Another possibility is that IFITM3 elicits two opposing effects on the NTCP-HepG2 cells by facilitating viral entry through interaction with NTCP and by restricting the virus release from the endosomes as shown for IAV. This could also explain why the effects of IFITM3 were not consistent across all cell lines and viruses since the relative importance of the two opposing factors might be different in each case. Nevertheless, it seems paradoxical that an interferon-responsive factor such as IFITM3 does not restrict, but supports, HBV/HDV infection. However, recent studies showed that other viruses, such as SARS-CoV-2 and human cytomegalovirus (CMV), also hijack IFITM proteins to ensure efficient virus infection [57,58]. Additionally, a recent study identified IFITMs as infection-promoting entry factors of the human coronavirus OC43, although with an unknown mechanism [59]. It is tempting to speculate that HBV and certain HDV strains have evolved similar traits. Since our experiments were performed on naive liver cells, a scenario was used in which cells were confronted with the virus for the first time. As a hypothesis, HBV and HDV might hijack IFITM3 in endosomes after successful endocytosis to prevent degradation in an early infection period, whereas in a later phase of infection, the suppression of the host’s IFN response would be beneficial for the completion of the viral replication cycle. Taken together, IFITM3 may be regarded as a kind of enabling or promoting factor for in vitro HBV/HDV infection. Future experiments will be needed to figure out the exact mechanism behind this effect. Considering the clinical relevance of the presented data, the blocking of IFITM3 could be a possible way of limiting the HBV and HDV infection of hepatocytes. Studies have identified the mTOR inhibitor rapamycin and the antimycotic drug amphotericin B as inhibitors of the IFITM3-mediated restriction of IAV infection, but the mode of action is complex and not yet completely elucidated. However, it must be considered that rapamycin or amphotericin B treatment would increase the susceptibility to other viral infections such as IAV [60,61].

## 5. Conclusions

IFITM3 was identified as a novel co-factor of NTCP that significantly affects in vitro infection with HBV and HDV in NTCP-expressing hepatoma cells and PHHs. Based on the MYTH and co-IP experiments performed in this study, this effect is proposed to rely on direct protein–protein interaction between IFITM3 and NTCP, even though more experiments are needed to establish at which stage of the infection process this PPI is critically involved.

## Data Availability

Not applicable.
